# Biophysical assessment of the molecular mechanisms of Tau aggregation and its role in Alzheimer's disease

**DOI:** 10.1002/pro.70635

**Published:** 2026-05-26

**Authors:** Joshua T. Skrehot, Dmitry Kurouski

**Affiliations:** ^1^ Department of Biochemistry and Biophysics Texas A&M University College Station Texas USA; ^2^ Department of Chemistry Texas A&M University College Station Texas USA

**Keywords:** Alzheimer's disease, fibrils, oligomers, Tau, post translational modifications

## Abstract

Alzheimer's disease (AD) is characterized by the intracellular aggregation of the microtubule‐associated protein Tau. While the presence of large, insoluble neurofibrillary tangles has long been the primary focus of this research, a paradigm shift in the field now highlights smaller, soluble oligomers as the more neurotoxic Tau species leading to neuronal death and cognitive decline. This leaves the important and ill‐understood question of what molecular events lead to the conversion of healthy, functional Tau into these toxic oligomers. This review addresses the knowledge gap by investigating existing literature on the upstream mechanisms responsible for the onset of neurodegeneration and connecting it to disease pathogenesis. By synthesizing evidence from molecular biophysics, cellular biology, and neuropathology, this review summarizes the most recent understanding of factors contributing to pathological Tau aggregation, including post‐translational modifications, lipids, and metal ions, among others. This review also discusses how neurotoxic Tau aggregates contribute to the onset of AD. By connecting these factors with findings from mammalian brain studies, this review establishes a comprehensive timeline of pathology that demystifies the transition from physiological Tau to toxic oligomers and links specific molecular triggers to the onset of neurodegeneration.

## 1 INTRODUCTION

Alzheimer's disease (AD) is the leading cause of dementia, a category of diseases affecting an estimated 50 million people globally, which is projected to increase to 75 million by 2030 and over 100 million by 2050 (Gustavsson et al., 2023; Tay et al., 2024). The financial burden of this is estimated to be over $1 trillion USD annually, with numbers on the rise as diagnoses increase (Wimo et al., 2023).

AD is dually characterized by the extracellular deposition of amyloid‐β (Aβ) plaques and the intracellular formation of neurofibrillary tangles (NFTs) composed of Tau (Jeong, 2017). While the accumulation of Aβ is a critical early event in AD pathogenesis, the severity of cognitive decline correlates more closely with the spreading and accumulation of Tau aggregates rather than Aβ plaques (Götz et al., 2013). Aβ and Tau pathologies do not exist in isolation, but act synergistically to drive the synaptic dysfunction and neuronal death that is observed in AD. While both proteins are implicated in AD, a recent paradigm shift has pointed the focus toward Tau instead of Aβ as a major target for therapeutic intervention and understanding the pathogenesis of the disease.

The aggregation of the microtubule‐associated protein Tau in the brain is a defining feature of AD as well as other neurodegenerative diseases (Al Mamun et al., 2020). While large NFTs have been the primary focus of research in the past, a growing body of literature points to smaller oligomers of Tau as the primary perpetrator of neuronal damage and cognitive decline. This review addresses the important question of what molecular events lead to the initial conversion of healthy, functional Tau into these toxic oligomers. Clear understanding of the earliest triggers of Tau aggregation is essential for developing effective treatments that can target AD during the early stages and slow or prevent its progression. This review will set itself apart by combining evidence across multiple perspectives, from molecular biophysics to cellular biology and human neuropathology, to construct a clearer picture of how Tau aggregation begins and progresses to its various neurotoxic forms.

Two primary areas of focus are the intrinsic properties of Tau and the role of the neuronal environment. Post‐translational modifications (PTMs), differences between isoforms, and factors such as the composition of cellular membranes can act as modulators of this process by influencing Tau's aggregation and stability (Meraz‐Ríos et al., 2010). This research can be tied to evidence from human brain samples that depict the progression of neurotoxic Tau oligomers and the onset of AD. The intended outcome is a comprehensive story that depicts the process of Tau pathology in a holistic manner, providing an understanding of how upstream triggers of aggregation can eventually lead to neurodegeneration and AD.

**FIGURE 1:**
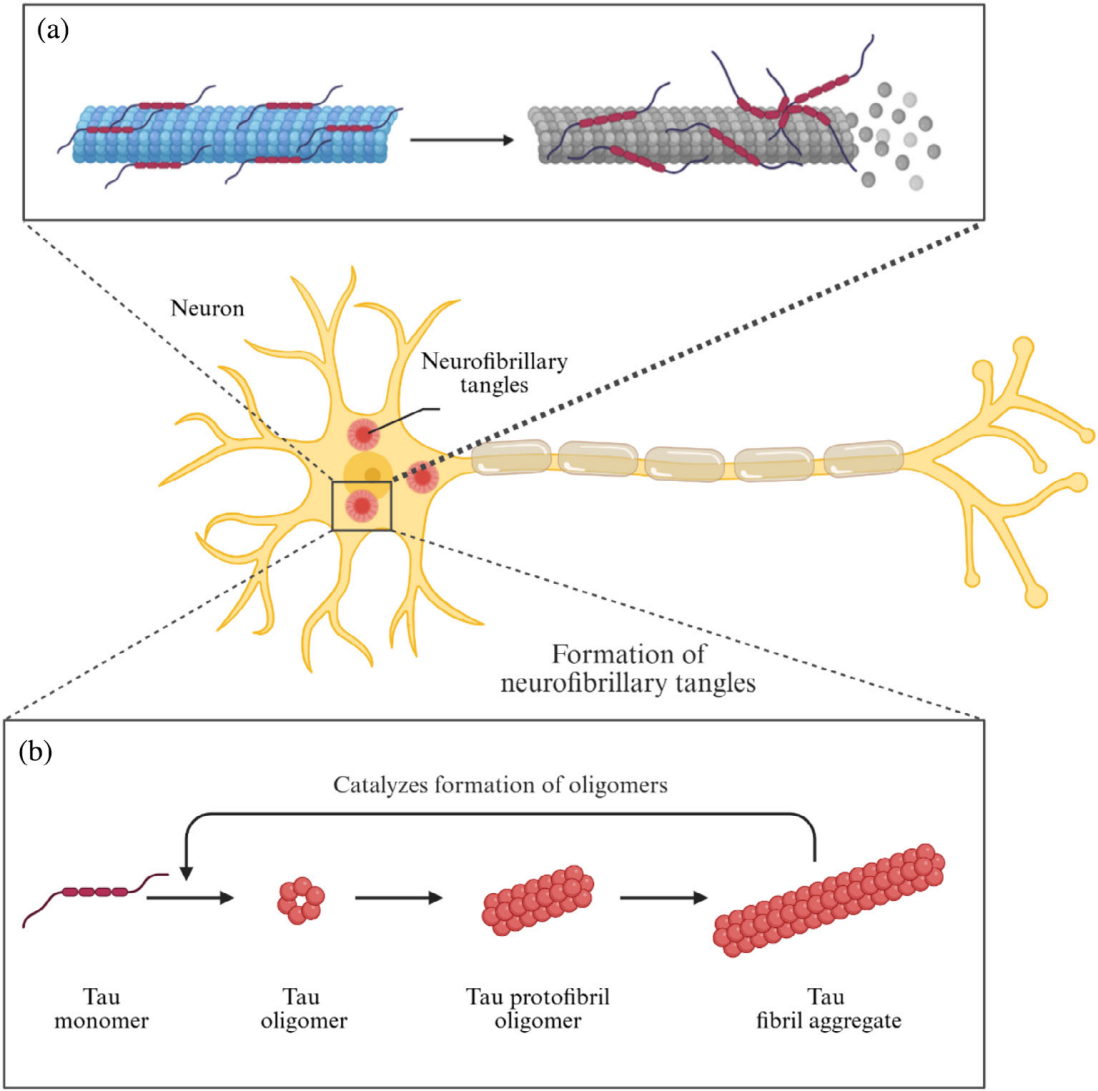
(a). Tau detachment from the microtubule is the first step toward protein self‐assembly. (b). Abrupt aggregation of monomeric Tau leads to the formation of oligomers that later propagate into protofibrils and fibrils capable of seeding Tau aggregation.

Tau is a protein that plays an important role in the assembly and stability of microtubules, the major components of the cellular cytoskeleton (Drechsel et al., 1992) (Figure 1a). In the case of AD and various tauopathies, Tau detaches from microtubules and aggregates into insoluble fibrils that form NFTs (Goedert et al., 1989) (Figure 1a). Although a portion of the existing literature suggests that NFTs are a main cause of neurotoxicity, this idea has been challenged by further research into earlier stages of Tau aggregation and subsequent loss of neurons and cognitive problems related to soluble, pre‐NFT forms of Tau (Figure 1b). These Tau oligomers have been found in the early stages of AD, long before NFTs form (Lasagna‐Reeves et al., 2012).

Under normal bodily conditions, Tau is soluble and does not typically aggregate on its own (Von Bergen et al., 2001). The process of Tau aggregation in vivo is not due to a single factor, but rather influenced by a complex network of variables. For instance, several isoforms of Tau exist in the brain as a result of alternative gene splicing, a process in eukaryotic cells that allows for variation in a gene's transcription (Bell‐Simons et al., 2024). Due to their varying structures, these isoforms aggregate differently, and their stability may be uniquely impacted by various cellular components like membrane phospholipids and cholesterol among other factors (Ali et al., 2025). Furthermore, Tau undergoes a variety of chemical changes known as PTMs. Phosphorylation is a frequently studied modification and is a key feature of tauopathies, but changes like ubiquitination and truncation among others also play a significant role (Morris et al., 2015). The timing of these modifications in relation to the formation of Tau oligomers is an important target in understanding the disease's progression. This review will combine analyze previous findings to provide a cohesive overview of the key factors that are believed to lead to the formation of neurotoxic Tau aggregates.

Summarizing, the main goals of this review are to (i) examine the various factors that trigger the aggregation of Tau, (ii) analyze the progression of Tau pathogenesis in the brain, including early‐ and late‐stage aggregates, (iii) develop a strong rationale between the pathological aggregation of Tau, the formation of oligomers, and their progression into larger supramolecular ensembles including amyloid fibrils and NFTs.

## 2 PHYSIOLOGICAL FUNCTION AND PATHOLOGICAL SELF‐ASSEMBLY OF TAU

**FIGURE 2:**
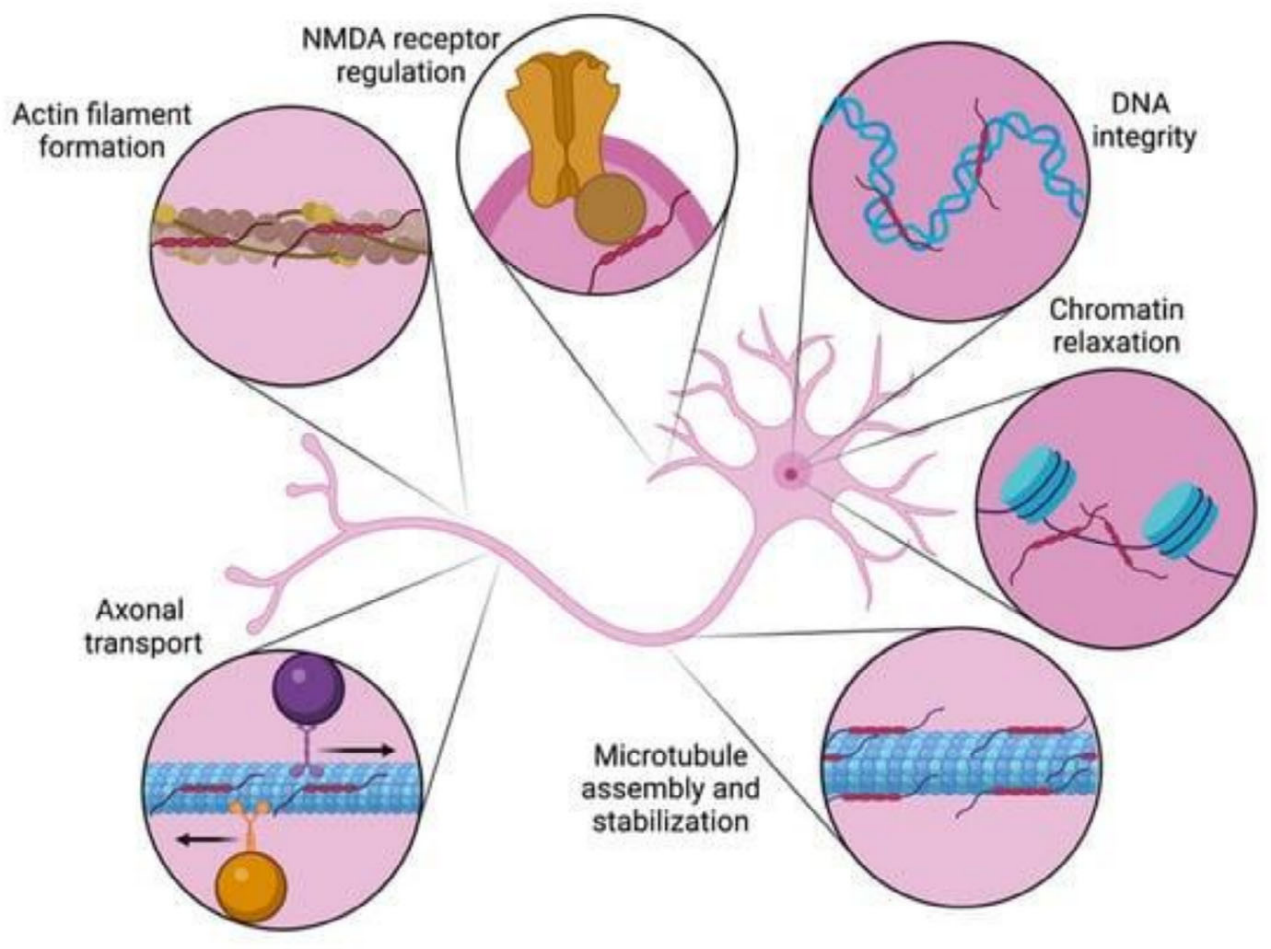
Tau's diverse physiological roles in the neuron. Initially characterized as a protein required for microtubule assembly and stabilization, it is now recognized that Tau has roles in multiple neuronal compartments. Along the axon, Tau is involved regulating in bidirectional transport as well as actin filament formation. Nuclear roles include protecting DNA integrity and promoting chromatin relaxation. At the neuronal membrane, Tau's interactions with the N‐methyl‐D‐aspartate receptor regulate its signaling. Reproduced from Holper et al. (2022), https://doi.org/10.3390/ijms23137307.

Tau is a microtubule‐associated protein that is found primarily in neurons, but also in lower concentrations in certain glial cells like astrocytes and oligodendrocytes (Forrest et al., 2023). In its unaggregated form, Tau carries out its physiological role by binding and stabilizing microtubules in the cell to support the cytoskeleton and allow for the intracellular transport of cellular components (Wang & Mandelkow, 2016) (Figure 2).

**FIGURE 3:**
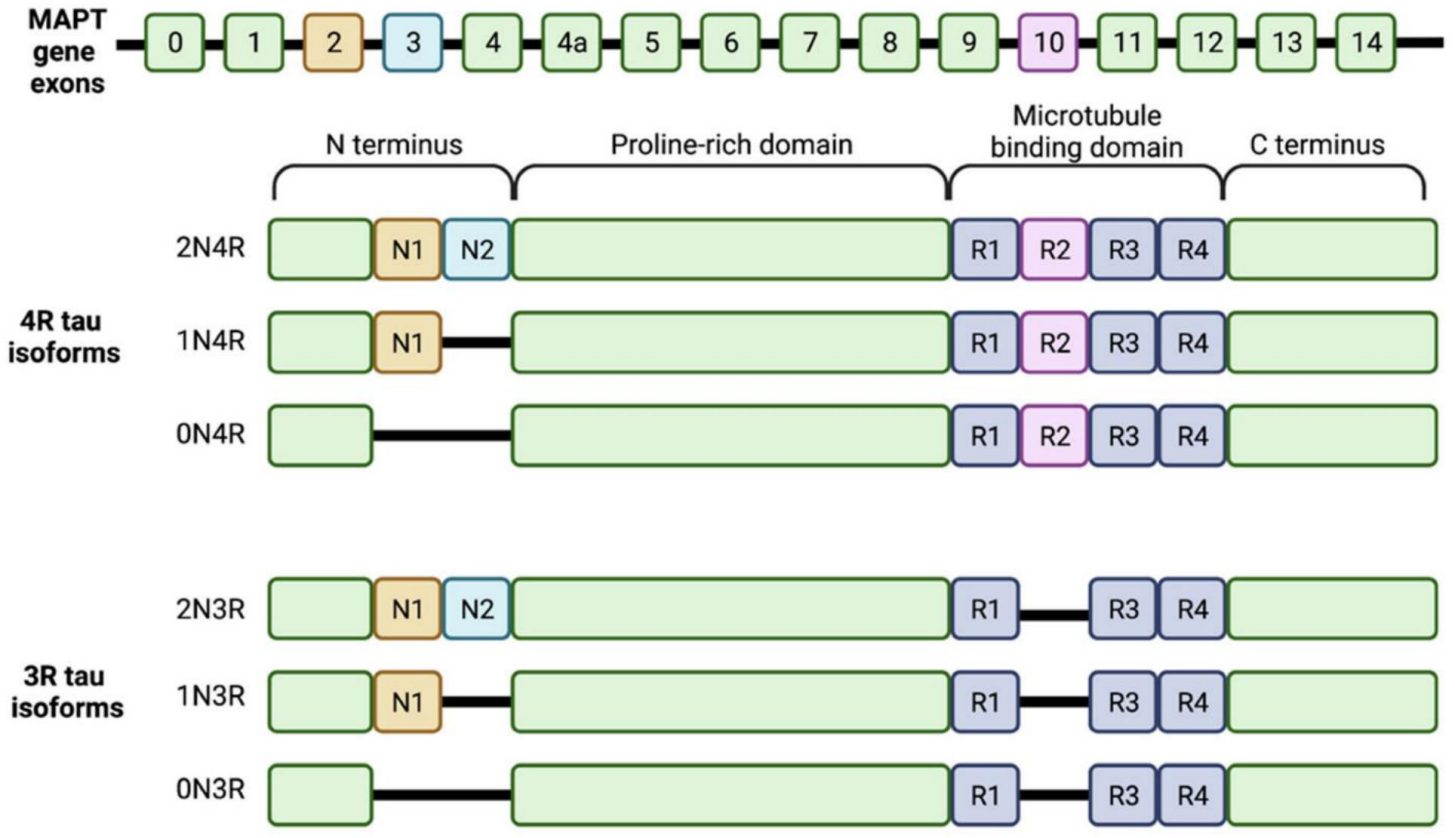
Six Tau isoforms present in the human brain produced via alternative splicing of the MAPT gene's 16 exons. Exons 2 and 3 encode the two possible N‐terminal inserts N1 and N2 (shown in orange and blue). Exon 10 encodes the second microtubule‐binding repeat (R2, shown in pink) in the microtubule‐binding domain. Alternative splicing results in six isoforms that vary by the number of N‐terminal inserts (0N, 1N, or 2N) and the presence or absence of R2 (four repeats [4R] isoforms or three repeats [3R] isoforms, respectively). Reproduced from Holper et al. (2022), https://doi.org/10.3390/ijms23137307.

Under physiological conditions, Tau is highly soluble in its monomeric form and lacks a defined secondary structure, remaining unfolded to enable flexibility to carry out its functions (Von Bergen et al., 2005). Structurally, it is made up of four distinct regions: the N‐terminal projection domain, a proline‐rich region, a microtubule‐binding domain, and the C‐terminal domain (Mandelkow & Mandelkow, 2012) (Figure 3). In the adult human brain, there are six different isoforms of Tau. The *MAPT* gene is alternatively spliced to produce the 0N3R, 0N4R, 1N3R, 1N4R, 2N3R, and 2N4R isoforms that are differentiated by the presence or absence of the N‐terminal projection domain and the number of repeats in the microtubule‐binding domain (Buchholz & Zempel, 2024). The most critical difference between these isoforms is whether their microtubule‐binding domain contains three repeats (3R) or four repeats (4R) of a specific binding sequence. These structural differences drastically change how the protein behaves. Functionally, the 4R Tau isoforms can grip the microtubules much more tightly and stabilize the cell's cytoskeleton more efficiently than the 3R isoforms (Goode et al., 2000). However, this extra gripping power comes with a tradeoff. The extra repeat section present in the 4R isoforms contains a specific amino acid (aa) sequence known as the PHF6* motif (Li & Lee, 2006). This sequence makes the 4R Tau highly prone to folding into rigid β‐sheets. Because of this, 4R Tau aggregates into toxic clumps much faster than 3R Tau.

A variety of biophysical techniques have been used to characterize the structure and morphology of Tau aggregates at different stages of pathogenesis. Atomic force and electron microscopy revealed the size and shape of these aggregates, while infrared and Raman spectroscopy were capable of probing the transition from disordered conformations (monomeric Tau) to β‐sheet‐rich structures commonly known as protofibrils and fibrils (Barth, 2007; Crowther, 1991; do Nascimento Amorim et al., 2024). Additionally, solid‐state NMR provided detailed mapping of the residues within the rigid core, and cryogenic electron microscopy has revealed near‐atomic structures of Tau filaments from the human brain, demonstrating that distinct tauopathies are defined by specific protein folds and filament characteristics (Daebel et al., 2012; Shi et al., 2021).

## 3 AGGREGATION TRIGGERS AND COFACTORS

The transition of Tau from a highly soluble, intrinsically disordered monomer into an insoluble and structured neurotoxic aggregate is a complex biophysical process driven by a multitude of internal and external cellular factors. In the healthy brain, Tau is heavily regulated to maintain its functional state. However, during the pathogenesis of AD and other neurodegenerative tauopathies, this homeostasis becomes imbalanced. The existing literature extensively documents that Tau aggregation is not a spontaneous event under physiological conditions but requires specific biochemical triggers. These triggers largely consist of PTMs, interactions with various cellular components, and changes in the cellular environment among other causes. By examining these molecular triggers in detail, a comprehensive understanding of the precise mechanisms that initiate Tau aggregation and AD pathogenesis can be established.

### 3.1 Post‐translational modifications

The functional state and structural conformation of Tau are predominantly regulated by a variety of PTMs, which are chemical alterations made to the protein after its translation from messenger ribonucleic acid (mRNA). Because Tau lacks a rigid tertiary structure, its exposed aa residues are highly vulnerable to chemical alterations. While physiological PTMs are essential for normal Tau function, the dysregulation of this system can become a trigger of dysfunction and aggregation.

#### 3.1.1 Phosphorylation

The most prominent and well‐studied chemical alteration that serves as an upstream trigger for Tau aggregation is phosphorylation. In a healthy brain, it is normal for the Tau protein to have a small, regulated number of phosphate groups attached to its structural surface at any given moment. These chemical tags are essential for healthy cellular function because they act as a molecular switch, constantly signaling Tau to bind the microtubule when dephosphorylated and release it when phosphorylated (Sengupta et al., 1998). This is because the microtubules are negatively charged and dephosphorylated Tau is positively charged, so phosphorylation causes Tau to become more negatively charged, creating repulsion from the microtubule (Hinrichs et al., 2012). This delicate balancing act allows the neuron to remain flexible and transport nutrients, mitochondria, and structural materials effectively down its axonal branches. It is when this system becomes imbalanced that Tau begins to aggregate.

The enzymes that phosphorylate and dephosphorylate proteins are called kinases and phosphatases, respectively, and they act at serine and threonine residues within Tau. In a diseased state, kinases may tip the scale by adding too many phosphate groups or phosphatases may fail to remove them at the necessary rate. The result is hyperphosphorylation, which causes Tau to remain detached from the microtubule, causing buildup and allowing it to aggregate with other phosphorylated Tau proteins (Morris et al., 2011). Recently, cryogenic electron microscopy and advanced imaging techniques have revealed that the specific sequence in which these phosphate groups are added acts like a template, predictably determining the three‐dimensional shape that Tau aggregates will eventually take as the pathology progresses, which has valuable implications for developing pharmacological treatments (Mammeri et al., 2024).

#### 3.1.2 Acetylation

Another PTM that has a functional role in the healthy brain, but can cause harm when dysregulated, is acetylation. Acetyltransferases carry out the transfer of a bulky acetyl group onto Tau's lysine residues. Tau is rich in lysine because it is a positively charged aa, allowing it to bind microtubules. When too many of these residues become acetylated, three specific issues occur. Acetyl groups have no net charge, so the neutralization of the positive charge when transferred to the lysine residue prevents Tau from binding microtubules. Additionally, this encourages the free‐floating Tau to fold into sticky, rigid shapes that are prone to aggregation. Beyond changing the protein's physical shape, acetylation is particularly devastating because it blocks the addition of ubiquitin, a protein that is added to Tau's lysine residues to mark the protein for degradation by a proteasome, clearing it out of the cell to prevent aggregation and harm (Min et al., 2010). This causes a rapid buildup of neurotoxic protein that leads to AD pathology.

The real‐world consequences of this specific tag have been observed in living models, where artificially mimicking the acetylation of lysine 280 was shown to exacerbate Tau‐mediated neurotoxicity in vivo in *Drosophila* (Gorsky et al., 2016). This establishes a link between excessive acetylation, neurodegeneration, and the cognitive deficits clinically observed in patients. Furthermore, research has shown that acetylation at highly specific “KXGS” motifs acts as a master switch that heavily dictates whether the protein will be safely cleared or toxically aggregated, highlighting the precise, highly localized nature of this chemical trigger (Cook et al., 2014).

#### 3.1.3 Oxidation

The human brain is a highly active, metabolically demanding organ that requires a large amount of oxygen and energy just to maintain baseline cognitive function. As a biochemical byproduct of this incredibly high energy demand, the brain is constantly exposed to harmful oxidative stress. This is most often caused by malfunctioning mitochondria, which begin to leak reactive oxygen species (ROS) into the intracellular environment as the brain naturally ages (Stefanatos & Sanz, 2018). High levels of oxidative stress lead to oxidation of Tau's cysteine residues within the repeat domains, causing them to form disulfide bridges within and between proteins, increasing the propensity to aggregate (Schweers et al., 1995). Disulfide bridges formed between Tau proteins create rigid dimers that act as sticky magnets that accelerate the recruitment of more free‐floating Tau to form larger aggregates. This process bypasses the slow, thermodynamically unfavorable early stages of aggregation and directly drives the rapid accumulation of protein that forms neurotoxic aggregates.

#### 3.1.4 Nitration

The oxidative stress introduced in the previous section is related to another destructive force known as nitrosative stress. The ROS generated by malfunctioning mitochondria often act synergistically with cellular nitrogen to form highly destructive nitrating agents, such as peroxynitrite, which can modify tyrosine residues on Tau (Horiguchi et al., 2003). This process physically alters the overall shape of the protein and impairs its functionality. By permanently preventing normal binding to microtubules, nitration causes the damaged, structurally altered Tau to form nitrated aggregates that pose a threat to the cell. While some structural alterations resulting from nitration initially seem to delay polymerization of Tau, the toxicity is due to its inhibitory effect on the ability of the protein to promote tubulin assembly, which compromises overall microtubule function and kills the neuron (Gendron & Petrucelli, 2009).

#### 3.1.5 Glycation and N‐glycosylation

As the brain ages, it begins to struggle with maintaining proper glucose metabolism and energy regulation. This metabolic depression leads to the harmful process of glycation, where sugar molecules permanently and non‐enzymatically attach themselves to lysine and arginine residues within the protein. While glycation alone does not initiate the microtubule detachment and aggregation of Tau, it enhances existing AD pathogenesis by stabilizing protein aggregates once they have already begun to fibrillize (Necula & Kuret, 2004). Once Tau begins to misfold, the attached sugars physically cross‐link the proteins, forming a thick, protective layer around the aggregate. This sugar coating makes the resulting aggregates incredibly stable and difficult for the cell's recycling system to break down and clear away.

Another similar, yet enzymatically driven modification is N‐glycosylation, which adds bulky carbohydrate chains to the asparagine residues of a protein. This modification has been found embedded in the brains of Alzheimer's patients and in vitro research has shown that reversing the glycosylation of Tau aggregates allows the protein to regain its functionality (Takahashi et al., 1999; Wang et al., 1996).

#### 3.1.6 Polyamination

Transglutaminase is a calcium‐dependent enzyme that primarily functions as a biological “glue.” Under healthy conditions, it facilitates the formation of covalent bonds between lysine and glutamine residues to stabilize protein structures, assist in cell adhesion, and support the integrity of the extracellular matrix (Wang et al., 2008). In AD brains, the activity of this enzyme has been found to be upregulated as the disease progresses (Johnson et al., 1997). Transglutaminase attaches small molecules called polyamines to the protein, creating permanent, indestructible covalent bonds between misfolded Tau. This cross‐linking process anchors the proteins together early in the disease timeline, locking them tightly into a toxic shape before the mature NFTs are fully formed (Singer et al., 2002). Because these covalent bonds are exceptionally strong and highly resistant to natural cellular recycling, enzymatic cleavage, and degradation, the cell's recycling mechanisms struggle to degrade and clear the aggregates out of the cell. This chemical cementing ensures that the small, toxic oligomers survive long enough to grow into permanent NFTs that damage the neuron.

#### 3.1.7 SUMOylation

SUMO proteins are small chemical tags that the cell uses to regulate healthy cellular activities and signal where natively unfolded proteins need to be transported (Dorval & Fraser, 2006). Under pathological conditions, SUMOylation stimulates the harmful hyperphosphorylation of Tau, signaling kinases to add destructive phosphate groups to the protein and force it to detach from the skeleton. Simultaneously, the bulky SUMO tag occupies the same lysine residue (K340) that undergoes ubiquitination for the cell to degrade and recycle dysfunctional Tau (Luo et al., 2014). This blocks degradation and causes the toxic, hyperphosphorylated protein to accumulate inside the neuron, contributing to the intracellular buildup that characterizes the later stages of AD.

#### 3.1.8 Protective PTMs

Fortunately, not all chemical tags are inherently harmful. The complex cellular environment also employs defensive modifications designed to keep Tau healthy, functional, and soluble. These protective modifications engage in a constant, dynamic balancing act against harmful PTMs and other causes of aggregation. In AD, this protective system eventually becomes overwhelmed.

- *O‐GlcNAcylation*: This defensive mechanism is a type of O‐glycosylation that involves adding a specific, simple sugar molecule to the serine and threonine residues on Tau that are usually vulnerable to harmful hyperphosphorylation. By physically occupying these highly vulnerable sites, O‐GlcNAcylation actively blocks the addition of destructive phosphate groups, serving as a natural protective cap that keeps Tau soluble, flexible, and fully functional, acting as a protective mechanism against AD pathogenesis (Liu et al., 2004). Specifically, this modification has been shown to heavily modulate and effectively halt the self‐aggregation ability of the fourth microtubule‐binding repeat of the XN4R isoforms of Tau (Yu et al., 2008).
- *Methylation*: The natural addition of methyl groups to Tau also serves a robust protective role. Lysine methylation is an endogenous modification that naturally depresses Tau's ability to stick to itself, significantly raising the thermodynamic threshold required for the protein to spontaneously clump together, delaying the onset of aggregation in a healthy human brain (Funk et al., 2014). Methylation also acts as competition for other harmful PTMs like acetylation, glycation, polyamination, and SUMOylation that occur at lysine residues. Maintaining a high, healthy level of methylation is therefore considered one of the most critical factors for sustaining long‐term neuronal health and preventing structural collapse (Balmik & Chinnathambi, 2021).
- *Ubiquitination*: In a normal brain, ubiquitination marks dysfunctional Tau from degradation to prevent aggregation and keep the cell healthy. This process signals the cell's proteasome system to safely and quickly clear away aging or damaged Tau before it can cause harm to the cell, though this system often becomes severely overwhelmed in AD pathology when there is a far greater volume of dysfunctional and aggregated protein (Li et al., 2022).
- *Prolyl isomerization*: This unique modification serves as a defensive mechanism by altering the conformation of Tau to facilitate the removal of harmful phosphate groups. Catalyzed by the enzyme Pin1 at the threonine 231 (T231) residue, this process twists the peptide chain from a *cis* to a *trans* conformation, exposing previously hidden phosphorylation sites. By making these sites accessible to phosphatases, this process drives the dephosphorylation of Tau, serving as a switch that restores the protein's ability to bind to microtubules and prevents aggregation in a healthy brain. However, this protective activity can be impaired by oxidative stress during the progression of AD (Martin et al., 2011).

The classification of specific PTMs as strictly protective or pathogenic is an oversimplification, which likely explains why many therapeutic approaches aiming to target protective PTMs have failed. Recent comprehensive mass spectrometry and cryogenic electron microscopy studies of human brain tissue have elucidated the complexity of Tau PTMs, identifying more than 95 distinct PTMs that dynamically interact and evolve over the course of the disease (Mair et al., 2016; Wenger et al., 2024; Wesseling et al., 2020). Though it is easy to evaluate them individually, Tau PTMs occur in a progressive, stepwise cascade that is highly dependent on the stage of AD (Wesseling et al., 2020).

Based on the summarized above findings, we can conclude that PTMs, lipid interactions, phase separation, and oxidative stress alter the dynamics of protein self‐assembly, which is an initiating event in the pathological self‐assembly of Tau. It should be noted that PTMs are the upstream triggers of protein aggregation rather than just consequences of it. However, other processes, including secondary nucleation, start playing an important role in the propagation of Tau oligomers into toxic fibrils.

#### 3.1.9 Proteolysis/truncation

Carried out by enzymes called proteases, proteolysis is the truncation of a protein into smaller fragments for a variety of reasons such as converting a protein to its active form or regulating its concentration. In AD, proteases worsen the pathological state by removing Tau's protective ends, exposing the highly sticky, aggregation‐prone inner core (Flores‐Rodríguez et al., 2015). These truncated fragments are unstable and act as potent, highly toxic seeds, rapidly recruiting healthy, full‐length Tau to misfold into growing, insoluble aggregates. Truncated Tau fragments taken from human brains have been found to interrupt the cell's recycling system and cause neuronal death in vitro (Corsetti et al., 2015). Gu et al. (2020) found that deletion of the first 150 or 230 aa enhanced Tau's site‐specific phosphorylation, self‐aggregation, and binding to oligomeric Tau isolated from AD brain tissue. Furthermore, Zhao et al. (2016) showed that P301L and D314E mutants of Tau were resistant to caspase‐2 cleavage, which prevented the mislocalization of Tau and glutamate receptors, restores synaptic function in cultured neurons, and rescues cognitive impairments of rTg4510 mice in a spatial reference memory test. For more detailed discussion of the effect of Tau truncation in the pathogenesis of AD, the readers are advised to read a fantastic review by Chu et al. (2023).

## 4 TAU‐LIPID INTERACTIONS

Beyond the internal chemistry of the protein itself, the physical environment of the cell plays a large role in triggering aggregation. The outer membrane of the cell and its internal organelles are made of lipid membranes. When free‐floating, unbound Tau interacts with specific, negatively charged lipid membranes, the protein's native structure is altered (Bok et al., 2021). It is thought that membrane surfaces may facilitate Tau aggregation by increasing the concentration of localized Tau and helping to neutralize charges that would normally keep the protein separate and prevent aggregation (Elbaum‐Garfinkle et al., 2010). When this physical contact neutralizes Tau's repulsive electrical charges, it physically forces the normally flexible repeat domain to fold into a rigid helical shape upon binding to lipid surfaces (Barré & Eliezer, 2006). This interaction acts as a direct catalyst to the aggregation process without the need for other chemical triggers, making the resulting protein–lipid complexes incredibly stable and highly toxic to the surrounding cellular environment (Ait‐Bouziad et al., 2017).

Interestingly, the specific type of lipid heavily dictates how the protein behaves. Experimental findings reported by Ali, Holman, et al. (2024) and Ali, Matveyenka, et al. (2024) show that the tubulin‐binding N‐terminal regions of Tau alter these lipid interactions, and while saturated fats can accelerate aggregation and increase toxicity, the presence of unsaturated fats can inhibit aggregation and decrease toxicity. Ali and co‐workers also found that the presence of cholesterol significantly increased the cytotoxicity of 0N4R and 1N4R fibrils to N27 rat dopaminergic neurons, an effect not observed for 2N4R. The study elucidated the following molecular pathways for Tau‐induced toxicity in cells. First, Tau aggregates enter cells via endocytosis and rupture endosomal membranes, triggering the ESCRT‐III repair complex (Chmp1) and de novo biogenesis of organelles (TFEB). Next, Tau aggregates induce the Unfolded Protein Response (UPR), significantly upregulating PERK, ATF6, and XBP1. Finally, Tau fibrils cause severe mitochondrial depolarization, which ultimately leads to cell death.

These findings show how sensitive Tau and other amyloidogenic proteins are to the brain's lipid environment and suggests that the specific makeup of lipid surfaces heavily influences the onset of the disease (Kurouski, 2023). Consequently, monitoring these protein–lipid interactions and utilizing them as potential biomarkers provides a valuable perspective in understanding the underlying mechanisms of AD (Kosicek & Hecimovic, 2013).

## 5 MAPT GENE MUTATIONS

While the vast majority of AD cases are considered to be sporadic and do not involve direct, inherited genetic mutations in the *MAPT* gene, studying inherited forms of dementia provides valuable information about how Tau can aggregate in specific cases. Genetic mutations have been found to cause Tau aggregation in two distinct ways. First, some mutations alter how the brain splices the gene, creating an unbalanced ratio of Tau isoforms and increasing the propensity for aggregation. Second, mutations within the gene can lead to the translation of dysfunctional Tau that struggles to carry out its function of binding and stabilizing microtubules, causing cellular harm and promoting aggregation (Wolfe, 2009). In a healthy state, Tau naturally folds into a protective shape that hides its sticky core from the outside. Mutations interfere with Tau's ability to form this protective shape, favoring extended conformations that misfold the protein and expose its sticky core to the surrounding environment (Pounot et al., 2023). This structural shift reduces the thermodynamic barrier to aggregation, increasing the rate at which it occurs.

## 6 PRION‐LIKE PROPAGATION AND CROSS‐SEEDING

A paradigm shift in modern neuroscience has revealed that misfolded Tau behaves similarly to an infectious prion, a misfolded protein that causes misfolding in healthy proteins that it interacts with. Rather than every single brain cell failing independently, Tau pathology physically spreads its toxic structural failures across the brain, leading to widespread neurodegeneration (Goedert et al., 2017). Small, highly mobile Tau aggregates called “seeds” are released from a cell and are endocytosed by a healthy neighboring neuron, with the specific structure of the seed dictating the severity and pattern of the ensuing aggregation (Goedert & Spillantini, 2017).

Once inside the new cell, this invasive seed acts as a template that recruits the healthy cell's normal, functional Tau and forces it to misfold, adopting the expected morphology long before mature NFTs form. This event has been observed directly at the synapses in human AD brains (DeVos et al., 2018). To travel efficiently, these seeds utilize the brain's transport pathways to bypass cellular defenses and invade healthy tissue, a mechanism modeled in *Caenorhabditis elegans* to reveal the genes critical for endolysosomal integrity (Sandhof et al., 2025). Furthermore, this spreading is accelerated by the presence of Aβ plaques, the other protein implicated in AD pathology. These plaques create a localized environment that acts as a catalyst, enhancing the initial recruitment and facilitating the aggregation of Tau seeds into toxic neuritic plaques (He et al., 2018). Gotz et al. (2011) showed that injection of Aβ1‐42 fibrils in the brain of P301L mutant Tau transgenic mice accelerated the formation of NFTs. This process, known as cross‐seeding, highlights the overlap between the two main pathologies of AD and suggests a unified cause that requires complex dual modulation strategies to successfully treat (Nam et al., 2025).

## 7 RNA, POLYANIONS, AND METAL IONS

Because Tau naturally has a strong positive charge, it naturally repels other Tau proteins, keeping them separated and floating freely. However, the brain is full of negatively charged molecules, like RNA and glycosaminoglycans. When free‐floating Tau encounters these polyanions, the molecules act as a physical scaffold. For example, RNA binds and neutralizes Tau's positive charges, forms disulfide bridges between cysteine residues in the 3R region, and allows protein monomers to rapidly bind together to form aggregates seen in AD (Kampers et al., 1996). The specific chemical features of these polyanions heavily dictate how fast the aggregates form and what specific conformational states they adopt, with highly sulfated carbohydrates, like heparan sulfates, heavily impacting the progression of neurodegeneration by stabilizing various neurodegeneration‐related proteins and inhibiting their proteolysis (Maïza et al., 2018; Montgomery et al., 2023).

Furthermore, an imbalance of naturally occurring metals in the brain, such as zinc, iron, and copper, can cause excess binding with Tau. This has been shown to increase levels of ROS, making a strong case for metal‐mediated Tau aggregation (Ahmadi et al., 2019). This binding has been found to alter the protein's physical shape and synergistically interact with other stress factors, like hyperphosphorylation, to promote rapid aggregation as well as coaggregation with α‐synuclein, a protein often associated with Parkinson's disease among other neurodegenerative diseases (Nübling et al., 2012). These metal‐protein interactions play a profound role in the disease cascade, driving the pathogenic aggregation of both Aβ and Tau (Kim et al., 2018).

## 8 MOLECULAR CROWDING, LIQUID–LIQUID PHASE SEPARATION, AND HYDROPHOBIC INTERACTIONS

The interior of the cell is a crowded environment, packed tightly with proteins, fats, and water among other components. Under conditions of cellular stress or extreme protein crowding, highly concentrated Tau monomers can undergo a biophysical phenomenon called liquid–liquid phase separation (LLPS). In LLPS, Tau proteins reach a critical concentration and spontaneously “de‐mix” from the rest of the fluid, naturally accumulating to form dense, liquid droplets within the cellular fluid (Wegmann et al., 2018). This phase separation creates a highly crowded environment in which proteins become so close together that the energy required to begin misfolding is lowered, which can initiate pathogenic Tau aggregation. Driven by localized hydrophobic interactions, these liquid droplets eventually lose their natural fluidity and harden into solid, toxic gels (Lin et al., 2021). These gels inevitably become the rigid seeds of NFTs implicated in neurodegenerative diseases (Boyko & Surewicz, 2022).

## 9 ENVIRONMENTAL PH AND ISOELECTRIC SHIFTS

The electrical charge and structural stability of Tau are heavily dependent on the pH of its surrounding cellular environment. 0N4R, the most common isoform of Tau, is cationic at physiological pH, possessing a high isoelectric point of ~9.5 (Montgomery et al., 2023). Because its net charge fluctuates based on the surrounding environment, subtle changes in pH act as a regulatory mechanism that can trigger aggregation. Specifically, histidine residues located near the C‐terminus of the microtubule‐binding repeats function as direct pH sensors for the protein. While these histidine residues maintain a positive charge and bind microtubules at pH <7.5, a shift to a more basic pH (>7.5) causes them to deprotonate. This deprotonation lowers Tau's binding affinity, causing the protein to detach from the cytoskeleton and aggregate in the cytosol (Alquezar et al., 2021). Shifts toward a more acidic environment drastically alter the physical morphology of this unbound Tau. In laboratory settings, exposing stable Tau‐phospholipid complexes to a slightly acidic pH (6.0–6.5) provides the necessary trigger to rapidly convert them into long, ordered filaments with an aggregation‐prone β‐sheet structure (Ait‐Bouziad et al., 2017). These pH‐dependent transitions dictate the solubility of the protein, showing that fluctuations in pH, whether basic shifts driving detachment or acidic shifts accelerating fibrillization, act as environmental catalysts for neurodegeneration.

## 10 AGGREGATION STRUCTURE AND MORPHOLOGY

The physical transition of Tau from a healthy, highly soluble protein into an aggregated, insoluble tangle is a complex, multistep process that notably involves changes in solubility and secondary structure.

### 10.1 Monomers

**FIGURE 4:**
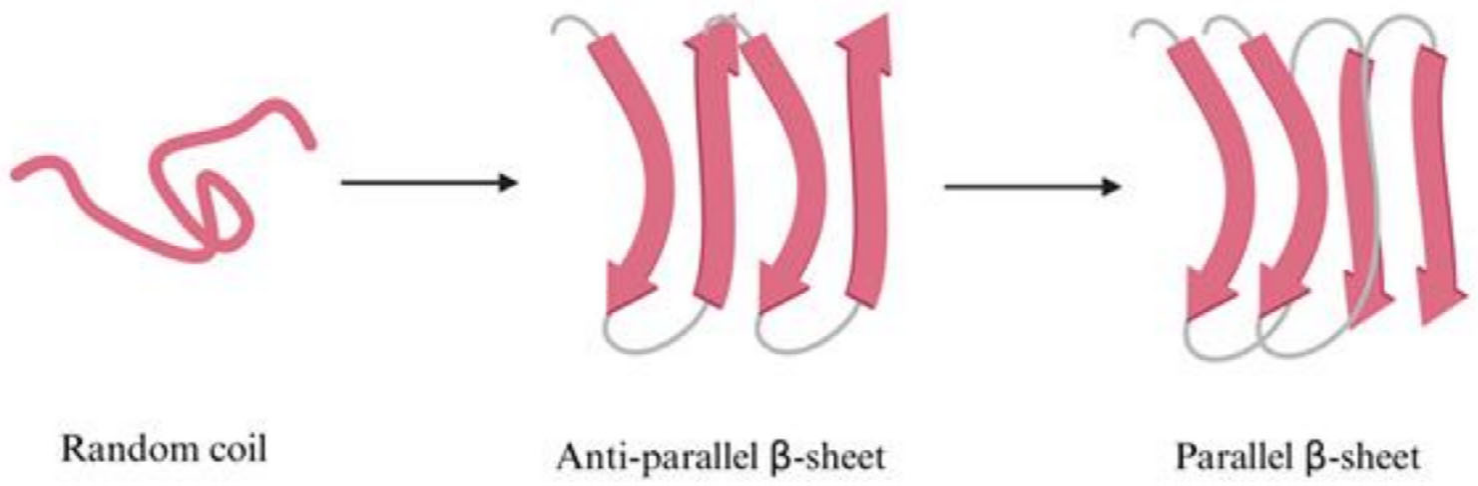
A scheme of transformations in the secondary structure of Tau observed during the aggregation.

In its healthy state, Tau exists predominantly as an intrinsically disordered monomeric protein. It behaves as a flexible random coil in the intracellular solution, lacking a rigid, well‐defined secondary structure (Figure 4). However, the microtubule‐binding repeat domain possesses a transient, low‐propensity β‐sheet character, and the protein can briefly acquire a α‐helix structure upon binding to lipid interfaces (Hernández et al., 2023; Wegmann et al., 2018).

### 10.2 Oligomers

Following mass detachment from the microtubule, the increased intracellular concentration of monomeric Tau begins a progressive self‐assembly process that results in the formation of oligomers, where Tau molecules cross‐link to form a structural template that facilitates further elongation (Figure 1b). This physical transition forces the native protein to rearrange its random coil structure into a more reactive antiparallel β‐sheet conformation (Schweers et al., 1995) (Figure 4).

### 10.3 Fibrils

As the aggregation process advances into the later stages, these oligomeric intermediates undergo a structural conversion as they stack and elongate into protofibrils and eventually into mature fibrils. During this maturation, the protein shifts from its earlier antiparallel β‐sheet conformation into a highly stable, parallel β‐sheet‐dominated structure (Daebel et al., 2012) (Table 1, Figure 4). Within these large NFTs, the secondary structure is entirely dominated by this rigid β‐sheet character, permanently trapping the previously flexible protein into a rigid mass that is difficult to remove from the cell.

**TABLE 1:** Morphology, solubility, and toxicity of different aggregation states of Tau.

Species	Morphology/structure	Solubility	Toxicity
Monomer	Single protein/disordered	Soluble	Non‐toxic
Oligomer	Granular clump/antiparallel β‐sheet	Soluble	High
Fibril	Long strand/parallel β‐sheet	Insoluble	Low

## 11 TOXICITY, NEURODEGENERATION, AND TREATMENTS

**FIGURE 5:**
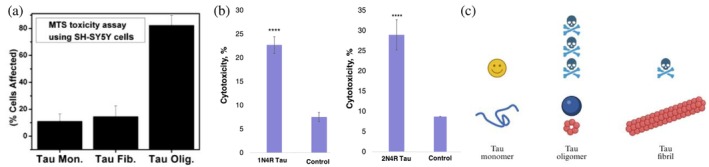
Tau oligomer toxicity shown by an MTS assay (a). Tau oligomers were toxic to SH‐SY5Y cells at the final concentration of 1 μM, while Tau monomers and fibrils were significantly less toxic. Reproduced from Lasagna‐Reeves et al. (2010), doi.org/10.1021/bi1016233. Histograms of lactate dehydrogenase assay showing cytotoxicity of 1N4R (left) and 2N4R Tau (right) aggregates formed in the heparin‐free environment without lipids (b). Black asterisks (*) indicate statistical significance of all samples relative to control. According to one‐way analysis of varience, NS is nonsignificant difference, *p < 0.05, **p < 0.01, ***p < 0.001, and ****p < 0.0001. Reproduced from Ali, Matveyenka, et al. (2024), https://doi.org/10.1021/acs.jpclett.4c01718. Relative levels of cytotoxicity for different aggregation states of Tau (c).

The progression of AD is tied to the shifting toxicity of Tau as it aggregates. Historically, researchers firmly believed that the large, highly insoluble, and microscopically visible NFTs were the primary Tau species killing brain cells in AD. However, modern scientific consensus has shifted, revealing that the solubility and size of Tau species dictate their toxicity. Monomeric Tau, in its healthy, highly soluble, and natively unfolded state, is generally considered non‐toxic and is essential for normal cellular function across different brain regions. However, as these monomers detach from the microtubules and aggregate into highly soluble intermediate species known as oligomers, they acquire highly neurotoxic properties. Granular Tau oligomers, representing an early sign of brain aging and AD pathology, exhibit high cellular mobility and toxicity due to their soluble nature and exposed β‐sheets (Maeda et al., 2006). These intermediate structures are now universally recognized as the dominant neurotoxic species driving the onset of AD (Cárdenas‐Aguayo et al., 2014) (Figure 5c).

Conversely, there have been varying reports on the toxicity of mature, completely insoluble fibrils, with Lasagna‐Reeves et al. (2010) finding them non‐toxic and Ali et al. (2025) finding them more toxic relative to monomers (Figure 5a,b). It is possible that they even serve as a protective cellular mechanism when the dying neuron attempts to safely convert highly toxic oligomers into inert fibrils, paradoxically highlighting the leading role of intermediate rather than mature Tau aggregates in neurodegeneration (Sahara et al., 2008). The toxicity of these intermediate aggregates explains the cognitive deficits seen universally across various tauopathies (Takashima, 2013).

**FIGURE 6:**
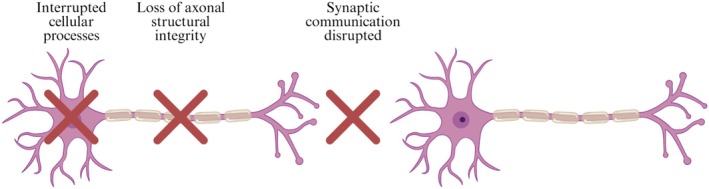
Mechanisms of neurotoxic effects of Tau aggregates.

The exact mechanisms by which soluble oligomers kill the neuron are complex and devastating, simultaneously disrupting a wide array of cellular processes ranging from genomic stability and energy production to cytoskeletal integrity and protein degradation (Niewiadomska et al., 2021) (Figure 6). Because of their small size, these oligomers easily travel down the cell's axonal branches and invade the synapses, serving as the primary toxic species at the communication points between neurons (Guerrero‐Muñoz et al., 2015). At the synapse, these oligomeric species disrupt the chemical signals necessary for memory consolidation and learning, heavily dictating neurodegenerative processes long before mature NFTs ever form (Ward et al., 2012). This synaptic interruption triggers a massive loss of function where Tau fails to stabilize the cytoskeleton, combined simultaneously with toxic aggregates that restrict axonal transport systems, starving the synapse of vital nutrients (Ballatore et al., 2007). Furthermore, these aggregates can embed themselves directly into the lipid membranes of the cell and its mitochondria. By doing this, they modify membrane ion conductance and trigger the activation of voltage‐gated calcium channels and NADPH oxidase (Esteras et al., 2021). These changes harm the integrity of the cell by causing an influx of calcium ions and sparking an increase of ROS, where mitochondrial oxidative stress further contributes to the pathogenic aggregation of even more Tau in a deadly positive feedback loop (Du et al., 2022). Ultimately, this cascade completely shuts down cellular energy production and kills the neuron, directly causing neurodegeneration as seen in AD.

As the brain's delicate and highly connected memory networks are progressively dismantled by these toxic species, the greater structure of the brain physically atrophies. This widespread misfolding, often synergistically exacerbated by the presence of Aβ, directly translates to the cognitive decline, memory loss, and personality degradation that define the clinical diagnosis of AD (Scheltens et al., 2021). Specifically, the progression of these clinical symptoms mirrors the spreading of Tau pathology throughout some of the brain's most vulnerable regions. In early AD, Tau‐driven neurodegeneration predominantly occurs in the entorhinal cortex and hippocampus, areas that are critical for learning and memory consolidation, which manifests clinically as a profound, progressive impairment in episodic memory (Langworth‐Green et al., 2023). As the disease advances, the propagation of neurotoxic Tau invades the neocortex, damaging neural circuits responsible for higher‐order cognitive functions. This structural collapse correlates directly with the loss of cognitive abilities, driving the executive dysfunction, language impairment, and behavioral degradation seen in late‐stage AD (Limorenko & Lashuel, 2022).

Following several failures of Aβ‐focused treatments and recognizing the centrality of Tau in driving the physical and cognitive destruction of the brain seen in AD, some modern clinical developments are focused on highly specific, Tau‐based treatments (Panza et al., 2016). Researchers are analyzing the Tau timeline in AD to design treatment strategies aimed at preventing aggregation and accelerating the clearance of aggregates (Götz et al., 2012). One cutting‐edge strategy involves the design of custom Tau aggregation‐inhibiting peptides, which are carefully crafted, non‐natural molecules designed to bind directly to the exposed, sticky core of the misfolded protein. By acting like a chemical cap, these peptides freeze the growth of the toxic fibril entirely and offer massive therapeutic potential (Aillaud & Funke, 2023).

Other clinical efforts are also actively testing small‐molecule drugs that inhibit the specific kinases responsible for the initial hyperphosphorylation of the protein, aiming to stop the primary aggregation trigger before Tau ever detaches from the microtubules. This includes utilizing agents like lithium to inhibit glycogen synthase kinase‐3, an approach that correlates with significantly reduced neurodegeneration in vivo in mice (Noble et al., 2005). Acknowledging the role of acetylation in Tau‐mediated neurodegeneration, clinical trials are actively testing repurposed drugs like salsalate to reverse this specific chemical tag in vivo in mice, showing recovered memory and stopping hippocampal atrophy (Min et al., 2015). Other pharmacological agents, such as the histone deacetylase 6 inhibitor MPT0G211, have been shown to successfully inhibit hyperphosphorylation and reverse aberrant acetylation in human and mouse cell lines, and reverse memory impairment in mice (Fan et al., 2018).

Beyond targeting specific chemical modifications and aggregation kinetics, researchers are exploring strategies to clear pathological Tau from the extracellular space, reduce overall protein expression, and restore damaged cellular structures. For instance, immunotherapies are currently a major focus of clinical trials. These approaches utilize engineered antibodies or synthetic peptide vaccines to target and clear toxic Tau seeds before they can spread their corruption to neighboring healthy cells. Both active and passive Tau immunotherapies report reduced pathology and significantly improved cognitive performance in mice models (Iqbal et al., 2016). Alongside clearance strategies, a genetic approach involves the targeted reduction of overall Tau levels using antisense oligonucleotides (ASOs). ASOs designed to target and degrade *MAPT* mRNA have recently received attention for their success in clinical trials, demonstrating reliability and safety with early signs of effectively reducing aggregated Tau within cerebrospinal fluid (Mummery et al., 2023).

Furthermore, because the detachment of Tau inherently compromises the neuronal cytoskeleton, researchers are investigating the direct stabilization of microtubules to combat AD pathology. The administration of microtubule‐stabilizing agents, such as the compound epothilone D, aims to persevere through Tau's functional loss by reinforcing the cytoskeleton independently, reducing axonal dysfunction (Zhang et al., 2012).

Despite the diversity of ongoing pharmaceutical developments and clinical trials, there have been no robust breakthroughs in late‐phase trials that suggest a promising leap forward in disease benefits. This is likely a result of multiple challenges, including the complex dual pathology of the disease, a lack of complete understanding of its underlying mechanisms, difficulty with drug delivery across the blood–brain barrier, and the common patient‐centered challenges of clinical trials, among other factors. Ultimately, the pursuit of an effective treatment for AD likely requires a multifaceted approach that comprehensively addresses the complex biology of Tau. The strategies highlighted above represent the foundational pillars of modern, Tau‐centric drug development. However, the valid therapeutic targets are by no means limited to these methods. While no therapy directly targeting Tau has achieved full clinical approval yet, many different therapeutic agents are currently advancing through human clinical trials, demonstrating that researchers are exploring a variety of avenues to address the spread of pathology. Because the toxic conversion of Tau is dictated by a diverse range of genetic, environmental, and biochemical influences, the exploration of these different avenues and the potential combination of multiple approaches offer the most promising path toward advancing AD treatment.

## 12 CONCLUSION

The pathogenesis of AD and other neurodegenerative tauopathies is linked to the structural and functional collapse of the microtubule‐associated protein Tau. The transition of Tau from a highly soluble, intrinsically disordered protein into rigid, neurotoxic aggregates is not driven by a singular event, but by a variety of factors. Through complex interactions between genetic vulnerabilities, environmental stressors, and biochemical processes, the native protein undergoes a neurotoxic structural and morphological evolution. These factors collectively impair Tau's physiological microtubule‐stabilizing function, leading to the generation of highly mobile, toxic oligomeric intermediates and the eventual formation of mature NFTs that crowd the cell. Ultimately, the progressive aggregation of these toxic species throughout specific brain regions drives the synaptic failure and neuronal death that characterizes the neurodegeneration observed in AD.

While the scientific community has made significant progress in elucidating the triggers of Tau aggregation, mysteries remain regarding the exact combinations of molecular events that initiate pathology in vivo. One of the greatest limitations in current research is the complexity of the environment within the human brain, which is difficult to accurately replicate in laboratory settings. For instance, while it is known that specific modifications, such as acetylation, directly inhibit physiological function and promote pathological aggregation, isolating these mechanisms ignores the dynamic interactions between dozens of simultaneous processes. Mapping of these processes in mice models has revealed that complex combinations of PTMs are far more relevant to disease pathogenesis than any single modification in isolation (Park et al., 2018).

Future research should focus on a more holistic approach, combining causal factors and replicating the natural brain environment more closely. Eventually, studies should begin to move away from single‐isoform models and explore how the six distinct brain‐specific isoforms interact and modulate the disease timeline. Furthermore, advancement of imaging techniques is necessary to fully decipher the structural progression of Tau aggregation and highlight new therapeutic targets. Ultimately, by bridging these remaining gaps, the medical community should hope to design universally effective pharmacological strategies capable of addressing the issue of Tau‐mediated neurodegeneration before any permanent cognitive erasure occurs and reversing existing pathology.

## AUTHOR CONTRIBUTIONS

**Joshua T. Skrehot:** Conceptualization; writing – original draft; writing – review and editing; visualization. **Dmitry Kurouski:** Writing – original draft; writing – review and editing; visualization; project administration; resources; supervision.

## Data Availability

Data sharing not applicable to this article as no datasets were generated or analysed during the current study.
